# Silencing of KCNA1 suppresses the cervical cancer development via mitochondria damage

**DOI:** 10.1080/19336950.2019.1648627

**Published:** 2019-07-27

**Authors:** Li Liu, Yumei Chen, Qingyuan Zhang, Changzhong Li

**Affiliations:** a Department of Obstetrics and Gynecology, Shandong Provincial Hospital Affiliated to Shandong University, Jinan, Shandong, China; b Department of Obstetrics and Gynecology, Wenzhou People’s Hospital, Wenzhou, Zhejiang, China; c Department of Neurology, Wenzhou People’s Hospital, Wenzhou, Zhejiang, China

**Keywords:** Voltage-gated potassium channel subfamily A member 1 (KCNA1/Kv1.1), cervical cancer, Hedgehog (Hhg), Wnt, Notch signaling pathway

## Abstract

Voltage-gated potassium channel subfamily A member 1 (KCNA1/Kv1.1) is an important component of type A potassium channels, which has been found to be involved in various tumors. This study aimed to identify the role of KCNA1 in cervical cancer and explore the related mechanism. The levels of KCNA1 in cervical cancer tissues and cell lines were examined by Western blot and qPCR. Cell proliferation and invasion were assessed by CCK-8 and transwell assays, respectively. Protein levels of Hedgehog (Hhg), Wnt and Notch were detected by Western blot. The mitochondrial capacity was examined by immunostaining with MitoTracker Red CMXRos. KCNA1 was highly expressed in cervical cancer tissues and cell lines, and correlated with poor prognosis. In addition, depletion of KCNA1 suppressed growth, proliferation, migration and invasion of HeLa cells. Moreover, KCNA1 could regulate the Hhg, Wnt and Notch signaling pathways and cause mitochondrial dysfunction. The present study has demonstrated that KCNA1 is an oncogene excessively expressed in cervical cancer, and promotes tumor progression by regulating the Hhg, Wnt and Notch signaling pathways and the mitochondrial capacity. Therefore, our results provide a theoretical basis for the discovery of novel clinical treatment against cervical cancer.

## Introduction

Cervical cancer, arising from the cervix, is one of the most common malignancies among women worldwide [1], second only to breast cancer [2]. The incidence rate of cervical cancer is about 500,000 cases each year worldwide [3], and the number of new cases and deaths in China accounts for more than one quarter of the world [4]. At the same time, the age of onset of cervical cancer tends to be younger [5]. In addition, the mortality rate of cervical cancer ranks the first among female malignant tumors, therefore making cervical cancer one of the major diseases seriously threatening women’s lives [6]. There are many reasons for the incidence of cervical cancer, the most important one of which is human papillomavirus (HPV) infection [7]. Based on current difficulties in early prevention, early screening and early treatment of cervical cancer, related research on cervical cancer has always been the focus of research attention.

Voltage-gated potassium channel subfamily A member 1 (KCNA1/Kv1.1), encoded by the *KCNA1* gene in human, is an important protein involved in the shaker related voltage-gated potassium channel [8]. Generally, KCNA1 acts as a potassium selective channel through which potassium ions can pass through the electrochemical gradient, which is a critical step in the repolarization of the membrane [9]. KCNA1 is more studied in brain diseases, especially epilepsy. Mutations in KCNA1 have been demonstrated to cause episodic ataxia type 1 [10]. In recent years, KCNA1 has also been found to be closely related to the occurrence and development of tumors. Previous research identified that KCNA1 was downregulated in several tumor types, and might be associated with the aggressiveness of breast cancer [11]. However, currently there has been no study focusing on the function of KCNA1 in cervical cancer.

In the present study, we revealed the relationship between KCNA1 and cervical cancer, and explored the underlying mechanisms to provide a theoretical basis for the discovery of new clinical treatments against cervical cancer.

## Methods

### Patients and samples

From February 2015 to August 2018, 20 patients diagnosed with cervical cancer were recruited at Shandong Provincial Hospital Affiliated to Shandong University. The tumor tissues and the adjacent non-tumor tissues were obtained during surgery. Tissues were maintained at −80°C for further RNA extractions. All patients who participated in the study have signed written informed consent forms, and this study was approved by Shandong Provincial Hospital Affiliated to Shandong University.

### Cell culture

Cervical cancer cell lines (HeLa, SiHa and C-33 A) and normal cervical epithelial cell line Ect1/E6E7 were purchased from American Type Culture Collection (ATCC, Manassas, VA, USA). Ect1/E6E7 was cultured in Keratinocyte-Serum Free medium (Gibco, Carlsbad, CA, USA) supplemented with 0.4 mM calcium chloride, 0.05 mg/ml bovine pituitary extract, and 0.1 ng/ml human recombinant EGF. HeLa, SiHa and C-33 A were cultured in DMEM medium (Gibco, Carlsbad, CA, USA) with 100 μg/ml streptomycin, 100 U/ml penicillin (Gibco, Carlsbad, CA, USA) and 10% fetal bovine serum (FBS). A 37°C and 5% CO_2_ condition is supplied for the culture of all cells.

### Establishment of stable KCNA1 knockdown or overexpressed cell lines

The stable knockdown cells for KCNA1 was prepared through infection of either HeLa cell line using shRNA or overexpressing lentivirus particles (ABM, MC, Canada). Positive cells were selected by 10 μg/ml of puromycin, and the stable HeLa knockdown or overexpressed cell line was confirmed by real time PCR with more than 65% of mRNA decrease or increase compared to control group.

### RNA extraction and qRT-PCR

Trizol reagent (Invitrogen Life Technologies, Carlsbad, CA, USA) was used for extracting total RNA according to the manufacturer’s instructions. For cDNA synthesis, a reverse transcription kit (Fermentas, St. Leon-Rot, Germany) was used. For quantitative analysis of mRNA levels, SYBR-Green Master mix (Life Technologies, Carlsbad, CA, USA) was applied.

### Cell counting experiments

Cell growth was determined by counting the cell number and observing the cell status under the microscope. For counting the cells, single cell suspension was prepared by trypsin digestion and proper dilution. The cell suspension was mixed with a 0.4% trypan blue solution at 9:1 (final concentration 0.04%). Under the microscope, living cells are dyed in a colorless, transparent shape, while dead cells show a distinct blue color. Live cells and dead cells were counted separately in 3 minutes. Cells in three different wells were averaged.

### Cell proliferation

Cell Counting Kit-8 (APExBIO Technology LLC, Houston, TX, USA) was used for assessing cell proliferation. The trypsin digested cells were counted and plated into 96‐well plates (5,000 cells per well). 10 μL CCK-8 solution was added to each well and incubated for 1–4 hours. A microtiter plate reader (Quant BioTek Instruments) was used for the measurement of CCK-8 at 450 nm. Cells in three different wells were averaged.

### Cell migration

Cell migration was determined by Radius™ 24-Well Cell Migration Assay (Cell Biolabs, San Diego, CA, USA) according to the manufacturers’ instructions. Cells in three different wells were averaged.

### Cell invasion

Transwell inserts (8 μm pore size; Corning) coated with Matrigel (R&D Systems, Minneapolis, MN, USA) were used for measuring cell invasion. The lower chamber of the transwell plates were filled with 750 μL medium with 20% FBS and the upper chamber was filled with 200 μL cells suspended with medium without FBS. The cells on the lower surface of Transwell inserts were fixed and then stained by crystal violet. Image pro‐plus (Media Cybernetics, Inc., Bethesda, MD, USA) was used for counting the cell numbers. Cells in three different wells were averaged.

### Western blot

Anti-KCNA1 (SAB4501618, 1: 1,000), anti-β-actin (A1978, 1: 2,000), anti-Notch3 (SAB1404139, 1: 500), anti-Hhg (SAB1407419, 1: 1,1000) and anti-Wnt1 (SAB2102711, 1: 1,1000) antibodies were purchased from Sigma-Aldrich (St. Louis, MO, USA). The mitochondria monoclonal antibody LS-B7980-50 was purchased from LifeSpan BioScience (Seattle, WA, USA).

### Nude mouse tumor formation experiments

NOD/SCID mice were bred in an animal facility. All animal experiments were conducted using an Institutional Animal Care and Use Committee-approved protocol. HeLa tumor cells (1 × 10^6^) were suspended in 100 µL cold PBS and injected into nude mice. The animals were monitored daily until they developed signs of neurological deficits or became moribund, at which time they were euthanized and tumor mass removed. The survival time was calculated and tumor volumes were determined by the formula volume (mm^3^) = (length × width^2^)/2.

### Measurement of mitochondrial capacity

Live cell mitochondria were detected by MitoTracker Red CMXRos (Invitrogen Life Technologies, Carlsbad, CA, USA). Mitochondria monoclonal antibody was used to determine the expression of MT by immunostaining and Western blot. The expression of MT was also detected by qPCR.

### Immunohistochemistry (IHC)

IHC was performed using a Vectastain Elite kit (Vector Laboratories, Burlingame, CA). Primary antibodies including the human-specific mitochondria monoclonal antibody (1:50) and Vimentin (1:200). After slides were incubated with primary antibodies for 90 minutes at room temperature, appropriate biotinylated secondary antibodies (1:200) were applied and incubated for 30 minutes. The final signal was developed using the 3,3ʹ-diaminobenzidine substrate kit for peroxidase. IHC staining was assessed by combining the intensity and extent of immunopositivity. Anti-mitochondria antibody (ab92824, Abcam, 1:50) was used for the green staining and the blue staining indicated the nucleus with DAPI.

### Statistical analysis

Heatmap was created using Multi-Experiment Viewer, part of the TM4 Microarray Software Suite. Data were presented as mean ± S.D. SPSS 16.0 software (SPSS, Inc., Chicago, IL, USA) was used for all the statistical analyses. To analyze differences between two groups, two-tailed Student’s t test was performed. One-way ANOVA following a Bonferroni post hoc test was performed to compare data between multiple groups. p < 0.05 indicate an accepted statistical significance.

## Results

### KCNA1 is highly expressed in cervical cancer tissues and cell lines and correlates with poor prognosis

**Figure 1.**
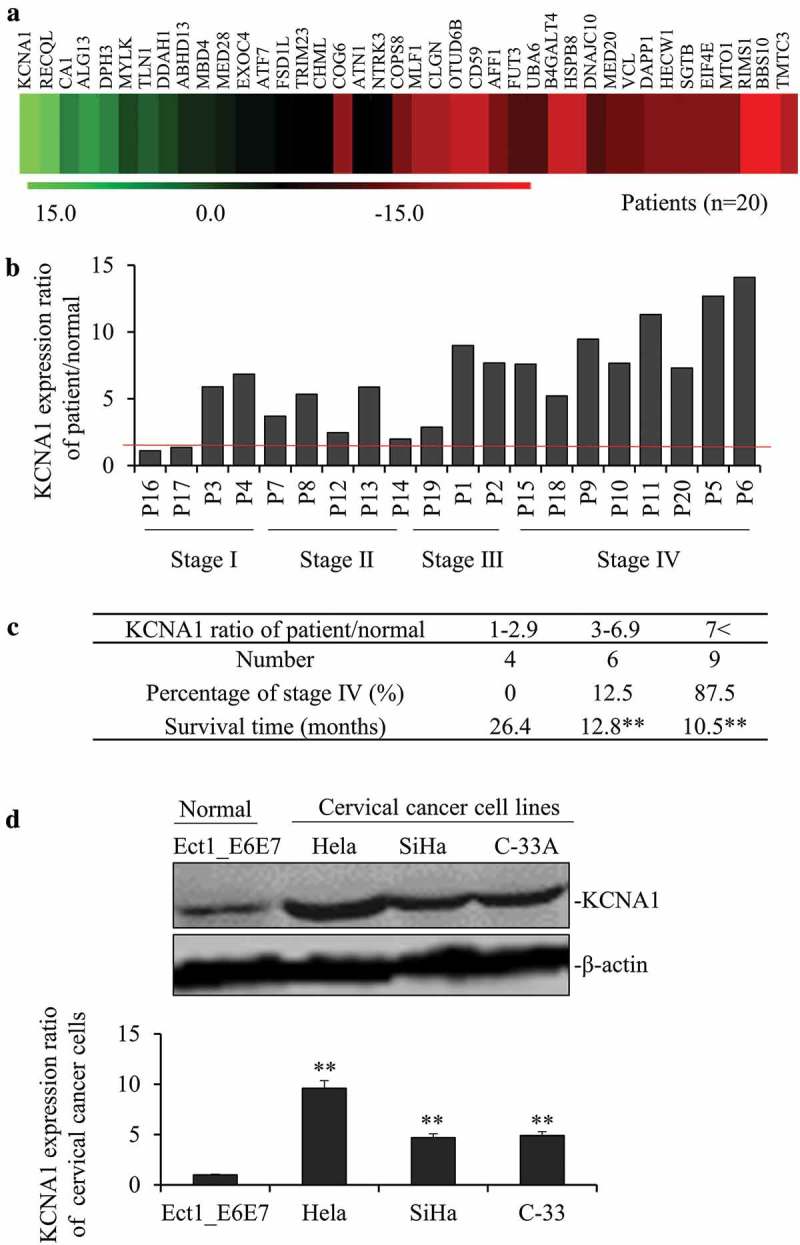
KCNA1 was highly expressed in cervical cancer tissues and cell lines and correlates with poor prognosis. a. The heat map showing the high-rich expression of KCNA1 in 20 cervical cancer patient tissues compared with adjacent non-tumor tissues (*p < 0.01*). b. The mRNA levels of KCNA1 were higher (ratio >2) in 17/20 representative cervical cancer patient tissues than in adjacent non-tumor tissues by PCR. c. The patient number and survival months were calculated with different KCNA1 expression (KCNA1 ratio from 1–2.9; 3–6.9 and >7) (**p < 0.01 compared with KCNA1 ratio 1–2.9 group). Meanwhile, the percentages of stage IV (%) in different KCNA1 expression (KCNA1 ratio from 1–2.9; 3–6.9 and >7) were calculated. d. The KCNA1 protein levels in 3 cervical cancer cell lines were normalized to the β-actin protein level and plotted. The data were shown as mean ± S.D. Quantitation by densitometry was shown on below (*P* < 0.01, n = 16, compared with normal human cervical epithelial cell line-Ect/E6E7). Three independent experiments were repeated.

To identify the role of KCNA1 in cervical cancer, we first examined the expression of KCNA1 in cervical cancer tissues from 20 patients. Compared with adjacent non-tumor tissues, KCNA1 was significantly upregulated in tumor tissues (Figure 1a). In addition, qPCR analysis indicated that the mRNA levels of KCNA1 were higher (ratio >2) in 17/20 representative cervical cancer patient tissues (Figure 1b). Next, we evaluated the correlation of survival months with different KCNA1 expressions. Our results revealed that excessive expression of KCNA1 was significantly correlated with short survival time and poor prognosis (Figure 1c). As the expression of KCNA1 increased, the survival time of patients was remarkably reduced. To further confirm these results, we detected protein levels of KCNA1 in cervical cancer cell lines (HeLa, SiHa, and C-33 A) and normal cervical epithelial cell line Ect1/E6E7 by Western blot (Figure 1d). Our results indicated that the three different cervical cancer cell lines showed significantly higher expression of KCNA1 than human normal cervical cell lines (p < 0.01). KCNA1 exhibited the highest expression in HeLa cells, therefore HeLa was used as the main experimental cell line in the following experiments. Taken together, we demonstrated that KCNA1 was significantly upregulated in cervical cancer tissues and cell lines, and correlated with poor prognosis.

### Knockdown of KCNA1 inhibits the cell growth of HeLa cells

**Figure 2.**
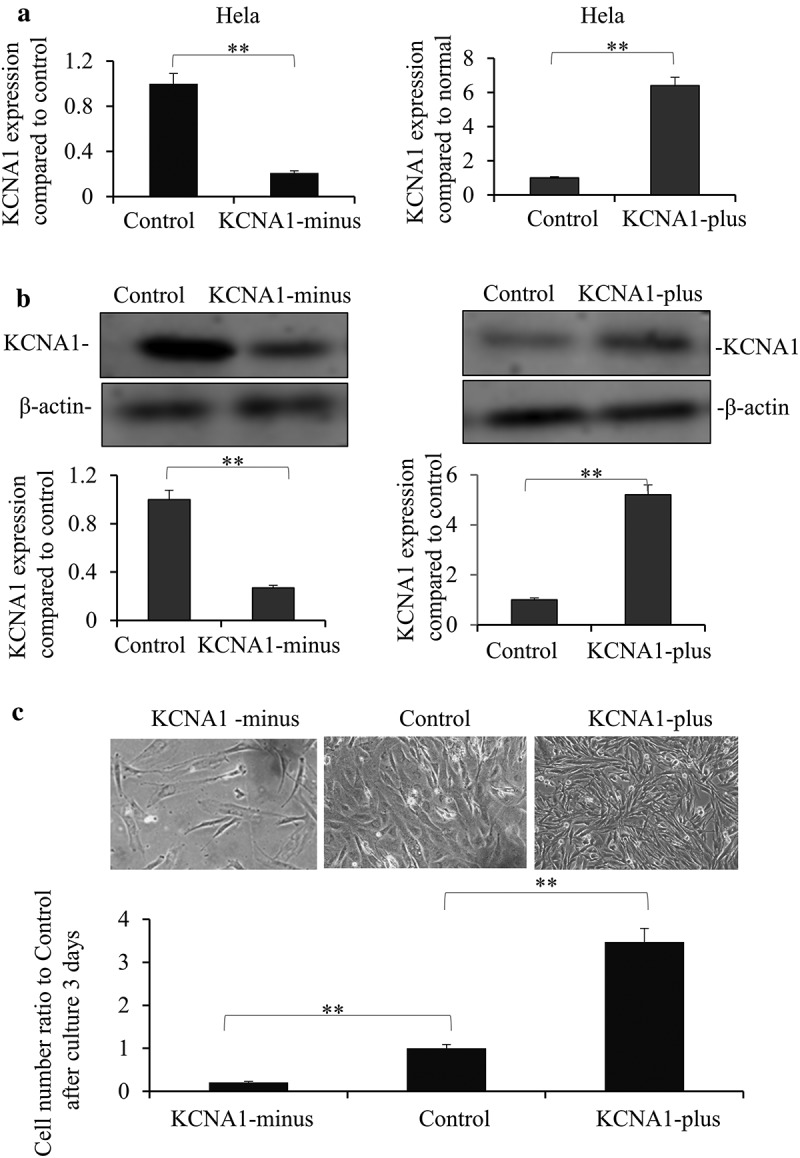
Knockdown and overexpression of KCNA1 affected the growth of HeLa cell line. Hela cells were transfected with Lenti-virus with KCNA1-plus or KCNA1-minus for 48 h. KCNA1 expression was detected by PCR (a) and Western blot (b) of three independent experiments. Quantitation by densitometry was shown on below (**P < 0.01, compared with control group, n = 6. Data are shown as mean ± S.D). c. The cell growth statuses were observed of KCNA1-minus, over-expressed and control Hela cells after culture for 3 days by microscopy. The original cell number were the same of 5 × 10 [4] in 6-well plate. Quantitation by counting the cell number was shown on below (**P < 0.01 compared to the control group, n = 6. Data are shown as mean ± S.D). Three independent experiments were repeated.

To identify whether KCNA1 affected cervical cancer cell growth, we first generated stable KCNA1 knockdown and overexpression HeLa cell lines by lentivirus. Our results showed that both the mRNA (Figure 2a) and the protein levels (Figure 2b) were successfully altered. Next, we examined growth of cells in these groups. By counting the cell numbers and observing the cell growth status, we found that knockdown of KCNA1 suppressed cell growth and overexpressing KCNA1 showed a reverse effect (Figure 2c). Therefore, our data suggested that KCNA1 regulated growth of cervical cancer cells.

### KCNA1 regulates the proliferation, migration and invasion of HeLa cells

**Figure 3.**
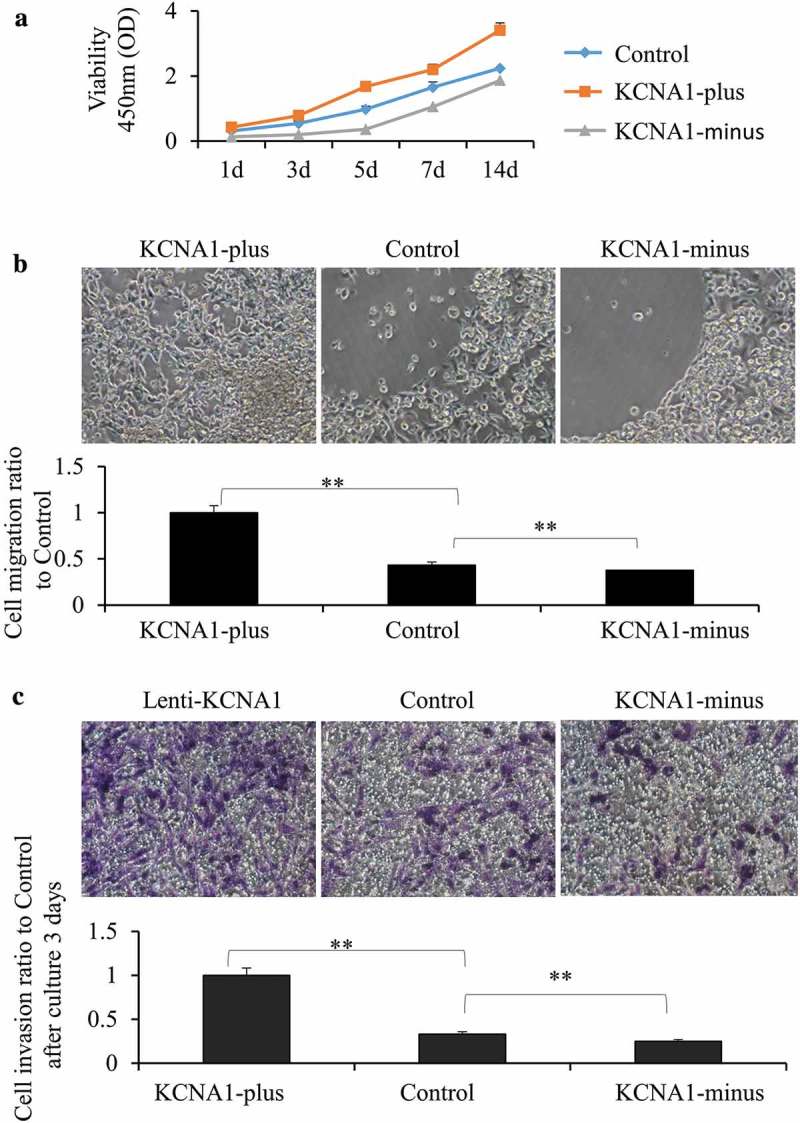
Knockdown and overexpression of KCNA1 affected the proliferation, migration and invasion of HeLa cells. a. CCK-8 assay was used to detect cell viability of Hela cells treated with Lenti-virus with KCNA1-plus or KCNA1-minus for 48 h. Hela cells were placed on 96-well plates (5 × 10^3^ cells/well) and incubated with fresh medium. Growth curves were detected. Points and range lines at different day (1, 3, 5, 7 and 14 days) represent mean and S.D. OD value was measured at 450 nm (**p < 0.01, n = 6). b. Migration kit assay with Lenti-virus with KCNA1-plus or KCNA1-minus for 48 h was tested. Migration of the cells to the blank area was visualized at 72 h with an inverted Leica phase-contrast microscope (9200 magnification). Quantitation was shown on below (**P < 0.01 compared to the control group, n = 6. Data are shown as mean ± SD). c. The relation of KCNA1 expression and invasion capacity was tested at 72 h after culturing cells by transwell assays. KCNA1-minus cells showed lower penetration rate through the membrane compared with control-shRNA and mock cells. Quantitation was shown on below (**P < 0.01 compared to normal cell line, n = 6. Data are shown as mean ± S.D). Three independent experiments were repeated.

To further investigate the function of KCNA1 in regulating the biological function of cervical cancer cells, we examined proliferation, migration, and invasion in the previous established stable KCNA1 knockdown and overexpression HeLa cells. CCK-8 assay revealed that knockdown of KCNA1 remarkably inhibited cell proliferation, while overexpressing KCNA1 exhibited a reverse trend (Figure 3a). In addition, cell migration assay showed that knockdown of KCNA1 suppressed, whereas overexpressing KCNA1 promoted, migration of HeLa cells (Figure 3b). Moreover, transwell assay demonstrated that the expression of KCNA1 was positively correlated with cell invasion. Knockdown of KCNA1 decreased, whereas overexpression of KCNA1 increased, the invasive ability of HeLa cells (Figure 3c). Taken together, our data indicated that KCNA1 modulated the proliferation, migration and invasion of HeLa cells.

### KCNA1 regulates the Hedgehog (Hhg), Wnt, and Notch signaling pathways

**Figure 4.**
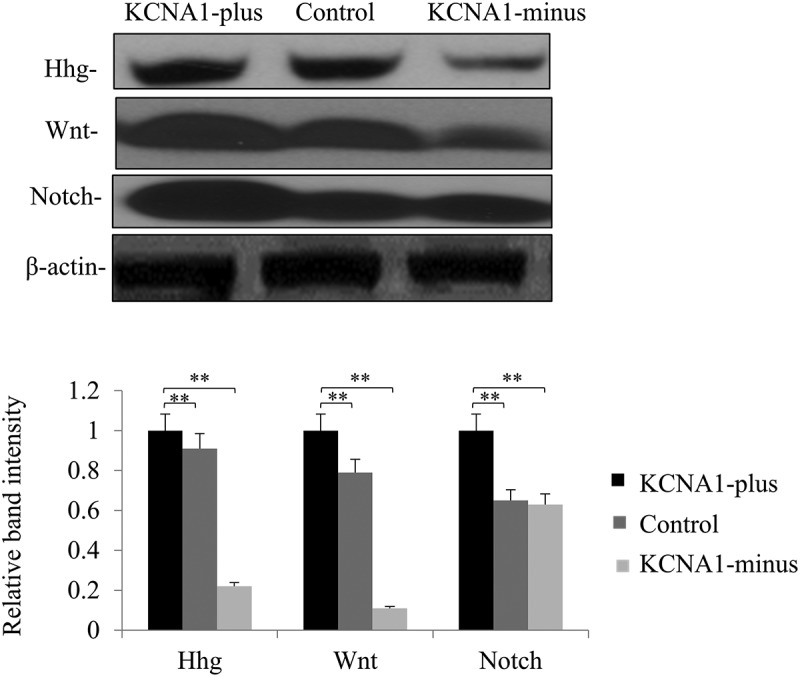
Relative signaling pathways responsible for the role of KCNA1 in affecting HeLa cell growth. Western blot analyses using the Hela cell extracts obtained from: control cell line vs Lenti-virus with KCNA1-plus or KCNA1-minus of Hela. The Hhg (DHH), Wnt (Wnt1) and Notch (Notch3) protein levels in HeLa cells were normalized to the β-actin protein level and plotted. The data were mean ± S.D. Quantitation by densitometry was shown on below (**P < 0.01, n = 16–18). Three independent experiments were repeated.

To further explore the mechanism involved, the activation of several signaling pathways that had been proved to be critical in cancer were examined, including Hhg, Wnt and Notch. The protein levels of Hhg, Wnt1 and Notch were detected in stable KCNA1 knockdown and overexpression HeLa cells, respectively (Figure 4). When compared with the control cells, knockdown of KCNA1 decreased the protein levels of Hhg and Wnt1, and overexpression of KCNA1 increased the protein levels of Wnt1 and Notch. Therefore, our results suggested that KCNA1 might affect the cellular function of cervical cancer cells by regulating the Hhg, Wnt and Notch signaling pathways.

### KCNA1 mediates cervical cancer development in vivo

**Figure 5.**
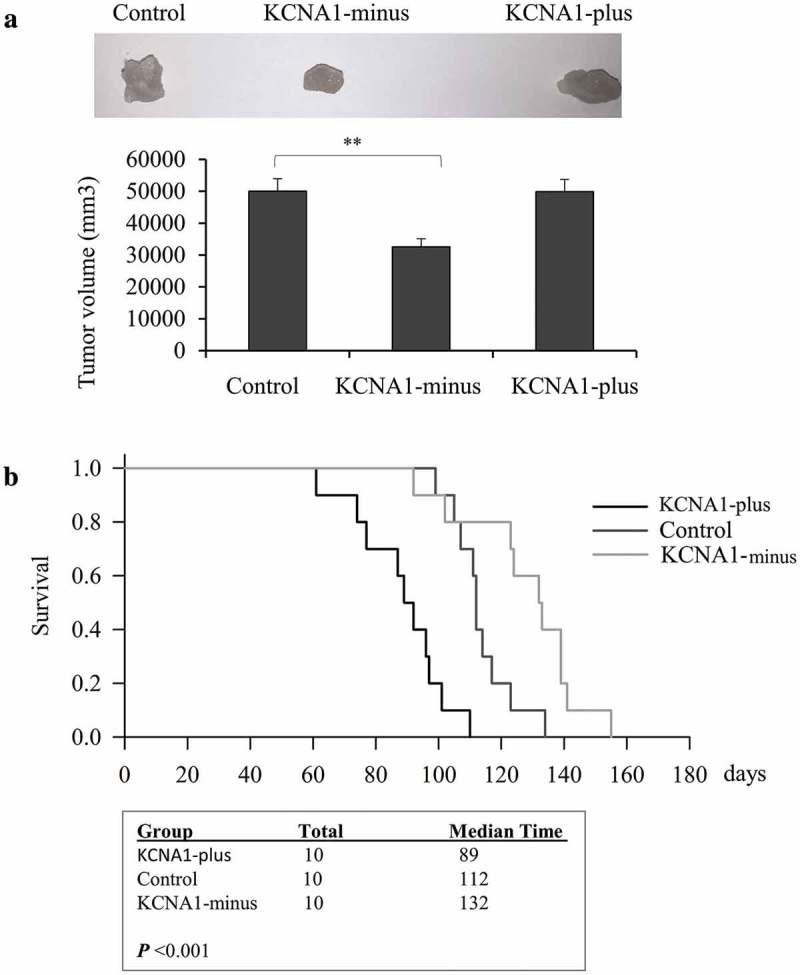
Knockdown and overexpression of KCNA1 affected cervical cancer development *in vivo*. a. The Hela cell line treated with Lenti-virus with KCNA1-plus or KCNA1-minus for 48 h were subcutaneous injected with 1 × 10 [6] cells. Tumor sizes were detected from different groups at two-months while all the mice were still survival but sick, moribund and skinny. Values are mean ± S.D. (**p < 0.01, n = 6). b. The Kaplan–Meier survival times were calculated after each mouse was dead (n = 10, **p < 0.01). Three independent experiments were repeated.

To verify our results from the *in vitro* experiments, we generated the *in vivo* model by subcutaneously inoculating the stable KCNA1 knockdown or overexpression HeLa cells into the bilateral flanks of nude mice. The tumor size and the status of the mice were monitored twice a week. Our data revealed that KCNA1 knockdown HeLa cells generated smaller tumors than control cells, whereas KCNA1 overexpressing HeLa cells formed larger tumors (Figure 5a). Next, we analyzed the survival time of mice in the three groups. Knockdown of KCNA1 significantly extended the survival time of the nude mice, whereas overexpression of KCNA1 shortened the survival time (Figure 5b). Taken together, our data indicated that KCNA1 regulated cervical cancer development *in vivo*.

### KCNA1 mediates the mitochondrial capacity in HeLa cells

**Figure 6.**
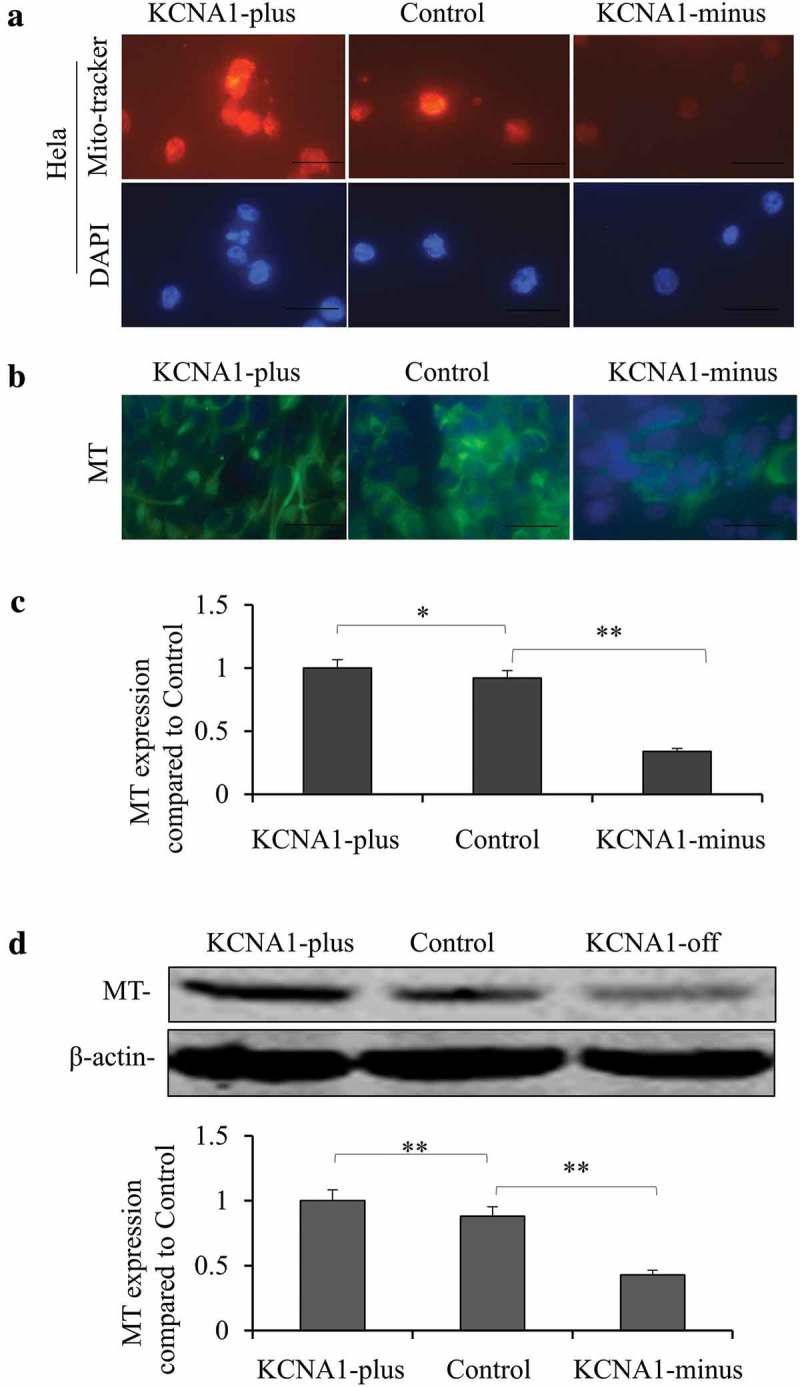
Knockdown and overexpression of KCNA1 affected the mitochondrial capacity in HeLa cells. b. Hela cell line treated with Lenti-virus with KCNA1-plus or KCNA1-minus for 48 h were stained with mito-tracker introduced. The positive staining presented the mitochondrial capacity of Hela cancer cell (bar = 200μm). b. Hela cell line treated with Lenti-virus with KCNA1-plus or KCNA1-minus for 48 h were stained with MT antibody. The positive staining of MT presented the mitochondrial capacity of Hela cancer cells (bar = 200μm). Anti-Mitochondria antibody was used for the green staining and the blue staining indicated the nucleus with DAPI. MT expression was detected by PCR (c) and Western blot (d). Quantitation by densitometry was shown on below (**P < 0.01, *P < 0.05 compared to untreated cell line, n = 16–18. Data are shown as mean ± S.D). Three independent experiments were repeated.

Emerging evidence has shown that mitochondrial dysfunction is closely related to the development of various tumors. Therefore, we investigated the function of KCNA1 in regulating the mitochondrial capacity. We first stained live cell mitochondria using MitoTracker Red CMXRos in KCNA1 knockdown and overexpression HeLa cells, respectively. Our results revealed that knockdown of KCNA1 decreased the positive staining and overexpression of KCNA1 showed a reverse effect (Figure 6a). Next, we examined the expression of mitochondrial marker (MT) in the three groups using the mitochondria monoclonal antibody. Our results showed that knockdown of KCNA1 decreased the expression of MT, whereas overexpression of KCNA1 increased the expression of MT (Figure 6b). In addition, qPCR and Western blot analyses showed that both the mRNA (Figure 6c) and protein levels of MT (Figure 6d) were upregulated in KCNA1 overexpressing HeLa cells, while downregulated in KCNA1 knockdown HeLa cells. Therefore, we concluded that KCNA1 mediated the mitochondrial capacity in HeLa cells.

## Discussion

There are many reasons for the incidence of cervical cancer, the most important and common of which is HPV infection[12]. As far as the diagnosis of cervical cancer is concerned, there are two problems at present[13]. First, the diagnostic method lacks accuracy. Detection of viral nucleic acids or tumor-associated markers cannot completely diagnose cervical cancer[14]. Second, there is no effective means to detect the degree of virus infection and the extent of cancer progression. It is difficult to predict the stage of the viral infection cycle before cancer[15]. Problems, such as the degree of tissue carcinogenesis, can only be obtained by retrospective studies of lesion tissues, which is not directly useful for cancer prediction or diagnosis[13]. The current solution is to use multiple detection methods to improve detection accuracy. In the present study, we found that KCNA1 was highly expressed in cervical cancer tumor tissues compared with adjacent non-tumor tissues. Therefore, KCNA1 might also be utilized as a novel diagnostic marker. In addition, we observed that excessive expression of KCNA1 was remarkably correlated with short survival time and poor prognosis. As the expression of KCNA1 increased, the survival time of patients was remarkably reduced. Therefore, detecting the expression levels of KCNA1 in cervical cancer could be used to predict tumor stages and survival time for patients.

KCNA1 is originally found to be involved in transmembrane potassium transport in the excitatory membrane, primarily in the brain and central nervous system, but also in the kidney[16]. Recently, it has been studied in various tumor types. A previous study revealed the tumor suppressive role of KCNA1, supported by its downregulated levels in breast cancer cells[17]. In addition, Bernard et al. reported that decreased expression of KCNA1 promoted the aggressiveness and invasiveness of primary breast tumors[11]. Besides, DNA methyl transferases and polycomb repressive complexes could decrease the expression of tumor-suppressive genes, including KCNA1, which might sustain the cancer inhibitory effects of KCNA1 [18–21]. However, there is also study indicating that high expression of KCNA1 in primary cervical cancers was associated with a need of complex bowel surgery and poor prognosis[22], which is convincing because it used cervical cancers primary tumors and their corresponding bowel metastases directly obtained from patients. In our study, we demonstrated a tumor promoting role of KCNA1 in cervical cancer. As mentioned before, we first observed increased expression of KCNA1 in tumor tissues compared with adjacent non-tumor tissues. In addition, the *in vitro* experiments showed that knockdown of KCNA1 suppressed growth, proliferation, migration and invasion of HeLa cells, which contradicted previous research. Moreover, our *in vivo* nude mouse tumor formation experiments further indicated that KCNA1 knockdown HeLa cells generated smaller tumors than control cells, whereas KCNA1 overexpressing HeLa cells formed larger tumors. Also, knockdown of KCNA1 significantly extended the survival time of the nude mice, and overexpression of KCNA1 shortened the survival time. Therefore, a new possibility for the role of KCNA1 in tumors is proposed in this paper. Our results contradict many previous results, which might be attributed to different cancer types used in experiments. The observation that KCNA1 is upregulated in cervical cancer tissues is more likely to trigger an oncogenic state, rather than simply be the result of a compensatory mechanism to maintain cellular homeostasis.

Various signaling pathways are involved in tumorigenesis and tumor development. Previous studies have found that Hhg signaling pathway is abnormally activated and subsequently participates in the development of cervical cancer [23]. The Hhg signaling pathway inhibitor cyclopamine inhibits the proliferation of cervical cancer cells by inducing apoptosis [24,25]. Therefore, Hhg signaling pathway may be regarded as a new target for the treatment of cervical cancer. In addition, the differential activation of Wnt and Hedgehog signaling pathways and their signal interaction have been demonstrated to play an important role in the development of cervical cancer [26]. Our results found that knockdown of KCNA1 significantly decreased the protein levels of Hhg and Wnt1, and overexpression of KCNA1 increased the protein levels of Wnt1. However, the expression of Hhg was not affected by overexpression of KCNA1. The content of KCNA1 in tumors is rich for the regulation of Hhg, and overexpression of KCNA1 does not increase the amount of Hhg, while knocking down KCNA1 has an important effect on Hhg. Furthermore, KCNA1 might be critical in regulating the levels of Wnt1.

Mitochondria are involved in the regulation of various cellular functions, and are implicated with many human diseases [27]. Cell signaling, metabolism, autophagy, aging and tumorigenesis are all associated with mitochondrial mass and activity[28]. In fact, mitochondrial biosynthesis and quality control are generally upregulated in cancer cells [29]. In the present study, we found that KCNA1 regulated the mitochondrial capacity of HeLa cells. Studies have shown that changes in mitochondria during apoptosis are related to intracellular potassium ion concentration [30], which regulates mitochondrial volume and reactive oxygen species. Abnormal intracellular potassium ion concentration can destroy transmembrane potential of mitochondrial inner membrane and induce Cytochrome C release [31]. In addition, KV1.1, KV1.3 and KV1.5 were found to exist in mitochondria and interact with BCL2-associated X, which in turn affected apoptosis [31]. Therefore, we speculate that KCNA1 alters the mitochondrial capacity by regulating the intracellular potassium ion concentration. Furthermore, KCNA1 might also function on cell apoptosis in cervical cancer.

## Conclusion

In conclusion, we have found that KCNA1 is highly expressed in tumor tissues of cervical cancer patients. KCNA1 affects the growth, migration and infiltration of cervical cancer cells and affects cell proliferation-related signaling pathways. In addition, KCNA1 influences the mitochondrial function. Therefore, our study suggests that KCNA1 might be utilized as a potential and novel diagnostic and therapeutic target for cervical cancer.
